# Comparison of strategies for substantiating freedom from scrapie in a sheep flock

**DOI:** 10.1186/1746-6148-5-16

**Published:** 2009-04-30

**Authors:** Benoit Durand, Marie-José Martinez, Didier Calavas, Christian Ducrot

**Affiliations:** 1 Unité d'épidémiologie, Afssa-Lerpaz, 23 avenue du Général de Gaulle, 94706 Maisons-Alfort, France; 2 Unité d'épidémiologie animale, UR346, Inra, 63122, St Genès Champanelle, France; 3 Unité d'épidémiologie, Afssa-Lyon, 31 avenue Tony Garnier, 69364, Lyon Cedex 07, France

## Abstract

**Background:**

The public health threat represented by a potential circulation of bovine spongiform encephalopathy agent in sheep population has led European animal health authorities to launch large screening and genetic selection programmes. If demonstrated, such a circulation would have dramatic economic consequences for sheep breeding sector. In this context, it is important to evaluate the feasibility of qualification procedures that would allow sheep breeders demonstrating their flock is free from scrapie. Classical approaches, based on surveys designed to detect disease presence, do not account for scrapie specificities: the genetic variations of susceptibility and the absence of live diagnostic test routinely available. Adapting these approaches leads to a paradoxical situation in which a greater amount of testing is needed to substantiate disease freedom in genetically resistant flocks than in susceptible flocks, whereas probability of disease freedom is *a priori *higher in the former than in the latter. The goal of this study was to propose, evaluate and compare several qualification strategies for demonstrating a flock is free from scrapie.

**Results:**

A probabilistic framework was defined that accounts for scrapie specificities and allows solving the preceding paradox. Six qualification strategies were defined that combine genotyping data, diagnostic tests results and flock pedigree. These were compared in two types of simulated flocks: resistant and susceptible flocks. Two strategies allowed demonstrating disease freedom in several years, for the majority of simulated flocks: a strategy in which all the flock animals are genotyped, and a strategy in which only founders animals are genotyped, the flock pedigree being known. In both cases, diagnostic tests are performed on culled animals. The less costly strategy varied according to the genetic context (resistant or susceptible) and to the relative costs of a genotyping exam and of a diagnostic test.

**Conclusion:**

This work demonstrates that combining data sources allows substantiating a flock is free from scrapie within a reasonable time frame. Qualification schemes could thus be a useful tool for voluntary or mandatory scrapie control programmes. However, there is no general strategy that would always minimize the costs and choice of the strategy should be adapted to local genetic conditions.

## Background

Scrapie is a neuro-degenerative transmissible disease, known since the 18^th ^century, that affects small ruminants and belongs to the group of transmissible spongiform encephalopathies (TSEs). Diseases of this group are characterized by the accumulation in the brain of an anomalous form of the prion protein (PrP), that induces nervous clinical signs leading to death. Bovine spongiform encephalopathy (BSE) and Creutzfeldt-Jakob disease are two other members of this group that respectively affect cattle and human. BSE was first described in 1987 [1]. In the following year, field studies allowed epidemiologists to identify its major transmission mode, meat and bone meal [2], and a first set of control measures were taken to break the transmission cycle. Ten years later, laboratory studies and epidemiological features showed that BSE agent was the probable cause of a new form of Creutzfeldt-Jakob disease in human [3,4], and a second set of control measures were taken, for public health protection. During BSE epidemic, European sheep were probably exposed to food contaminated by the BSE agent. Experimental studies have shown that sheep may be infected by BSE agent [5], and that the clinical signs are then identical to those of scrapie [6]. Furthermore, transmission experiments have shown the possibility of an horizontal transmission of BSE agent among sheep [7,8].

If sheep flocks have been exposed to BSE agent, a "sheep BSE" could thus exist and propagate silently, clinical cases being identified as scrapie cases [9]. The potential threat for public health induced by such a propagation led European animal health authorities to launch screening programmes for detecting BSE cases in small ruminants. To date, these programmes failed to detect BSE in sheep, but allowed to identify a TSE case in a goat, the agent signature of which could not be distinguished from that of BSE [10]. Distribution of pathological changes in the brain as well as western blot profiles allow to distinguish two scrapie types: classical scrapie and atypical scrapie. Epidemiological studies suggest that the aetiology and epidemiology of these two scrapie types are different, the infectious nature of atypical scrapie being uncertain [11]. The present study is specifically dedicated to classical scrapie, and the word 'scrapie' will be used below for 'classical scrapie'.

Genetic variations of susceptibility to scrapie have been described in numerous studies [12,13]. These variations are associated with the polymorphism of PrP gene, and individual susceptibility to scrapie may be characterized by three codons of this gene [14]. Despite available data are sparse, genetic susceptibility profiles to BSE in sheep seem similar to those of scrapie. Selection programmes have thus been launched in several European countries, that aim at eliminating the most susceptible alleles to decrease circulation of scrapie agent as well as the potential circulation of BSE agent in sheep [15].

Besides large abattoir or fallen stock screening programmes (which are very costly) and genetic selection programmes (the effects of which appear slowly in the general population), an intermediate approach centred on the flock may be proposed. As for other transmissible diseases, existence of a procedure to substantiate flock freedom from scrapie would allow defining objectively a subset of population considered as scrapie-free, a distinction that could be the basis of measures oriented towards public health or animal health. If a circulation of BSE agent in sheep flocks were demonstrated, such a qualification procedure would probably become mandatory, and form the basis of scrapie eradication plans, similar to those dedicated to other transmissible diseases such as tuberculosis or brucellosis.

However, a first particularity of scrapie is that unlike other transmissible diseases, no live animals tests are currently used in the EU for routine diagnostic, as tests are performed on brain samples. Therefore, scrapie can only be routinely diagnosed on slaughtered or dead animals. For other transmissible diseases, the status of a herd can be determined by testing all the animals living in that herd. But for scrapie, the status of a flock can only be based upon test results obtained from animals leaving the flock, that represent the remaining animals.

A second particularity of scrapie is that evidence of freedom from disease can not only be based on negative test results obtained on a sample of animals from the flock, but also on genetic data. Knowledge of the genotypes of the tested animals is necessary to interpret results of diagnostic tests: a negative result obtained from a genetically susceptible animal brings more evidence of scrapie freedom than a negative result obtained from a genetically resistant animal (because even if the flock were infected, this animal would probably have remained negative). Ideally, if live animals tests could be routinely used and if a full knowledge of flock genetics were available, animals of the most susceptible genotypes should be tested first to detect disease presence. However, in practice, it is not the case, and this situation leads to a paradox for flocks with a majority of genetically resistant animals: while in such flocks, the probability to be free from scrapie should be considered as high even if no animal has been tested for disease detection, performing diagnostic tests on the flock animals does not bring significant evidence of scrapie freedom as these animals are genetically resistant. Substantiating scrapie freedom through disease detection surveys would then be more difficult in resistant flocks than in susceptible flocks.

The objective of this study was to propose a framework for solving the preceding paradox, that allowed defining and comparing several practical approaches (termed below "qualification strategies") for substantiating scrapie freedom in a flock. These took into account scrapie specificities and differed according to how results of diagnostic tests were combined with genetic data to provide evidence of freedom from scrapie. Two sources of genetic data were considered: genotyping exams that may be performed on some of the flock animals, and flock pedigree (the set of parent-child relationships for the flock animals) that may also allow to derive genotype-associated probabilities for non-genotyped animals [16].

Feasibility of the proposed qualification strategies was quantified, in terms of probability for a scrapie-free simulated flock to be classified as such within a reasonable time. The corresponding durations were evaluated, as well as the associated costs: numbers of diagnostic tests and genotyping exams performed. The strategy for which the associated cost is the lowest was finally determined, as well as how this one varies according to the unitary costs of genotyping exams and diagnostic tests.

## Results

### Qualification strategies

A qualification strategy was defined by a specific combination of a testing scheme, a genotyping scheme and a level of information about flock pedigree. Two testing schemes were used: either no diagnostic test is performed, or all the culled animals are tested. Three genotyping schemes were considered: all the animals may be genotyped, leading to a full knowledge of the flock genetics; genotyping exams may be restricted to culled animals, or to founders animals. Founders animals are animals born in another flock, or animals of which one of the parents or both has been sold or culled (i.e. slaughtered at the end of its reproductive life). The third genotyping scheme thus supposes the pedigree is known: two information levels about flock pedigree were considered, the pedigree being either completely known or non-documented.

Combining the preceding testing schemes, genotyping scheme and information levels about flock pedigree led us to define six qualification strategies:

-two genotyping-oriented strategies in which full genetic knowledge is available (all animals having been genotyped), with (strategy A1) or without (strategy A2) diagnostic test performed on the culled animals,

-two diagnostic-oriented strategies in which a diagnostic test and a genotyping exam are performed on each culled animal, the flock pedigree being either known (strategy B1) or unknown (strategy B2),

-two pedigree-oriented strategies in which the flock pedigree is fully known and founders animals are genotyped, with (strategy C1) or without (strategy C2) diagnostic test performed on the culled animals.

Whatever the strategy, we assumed that, as soon as the flock is involved in a qualification process, rams used for mating are genotyped, and the farmer does not introduce any ram of the most susceptible genotype in his flock.

### Success or failure of the qualification process

In the simulations performed, two strategies always succeeded in a susceptible population (Table 1): a genotyping-oriented strategy (A1) and a pedigree-oriented strategy (C1). In both cases, genetic or pedigree knowledge is complemented by diagnostic tests on culled animals. Performing these diagnostic tests appeared essential, as the parallel versions of strategies for which no diagnostic test is performed (strategies A2 and C2) showed either a low success rate (A2) or a constant failure (C2). Performing diagnostic tests was however clearly not sufficient, as diagnostic-oriented strategies showed very low success rates (Table 1). Strategy B2, based on testing and genotyping of culled animals, always failed. Knowledge of the flock pedigree brought limited added value and the success rate remained low (12%) for strategy B1.

**Table 1:** Performances of six qualification strategies for substantiating scrapie freedom.

Population	Strategy	Qualification successes			
		Frequency (%)	Duration (years)^a^	Diagnostic tests^a^	Genotyping exams^a^
Susceptible	A1	50/50 (100%)	5.8	197	360
	A2	5/50 (10%)	8.8	0	464
	B1	6/50 (12%)	9.7	323	323
	B2	0/50 (0%)			
	C1	50/50 (100%)	5.8	196	138
	C2	0/50 (0%)			

Resistant	A1	47/50 (94%)	4.1	140	300
	A2	43/50 (86%)	5.1	0	335
	B1	0/50 (0%)			
	B2	0/50 (0%)			
	C1	47/50 (94%)	4.2	141	106
	C2	19/50 (38%)	8.3	0	186

^a^Mean values when qualification succeeds.

Results obtained in simulated flocks from a susceptible (30% susceptibility alleles and 10% resistance alleles, 50 flocks) and from a resistant population (10% susceptibility alleles and 30% resistance alleles, 50 flocks) according to the qualification strategy: (i) full genotyping with (A1) or without (A2) negative test results for culled animals, (ii) genotyping and negative test result for each culled animal with (B1) or without (B2) known flock pedigree, and (iii) genotyping of founders animals with known flock pedigree with (C1) or without (C2) negative test result for culled animals). The design prevalence is 1%.

In a resistant population, strategies A1 and C1 were again those for which success rate was the highest: 94% (Table 1). Their performances appeared very close, as both strategies failed in the same three simulated flocks. Performing diagnostic tests appeared less essential than in a susceptible population, as strategy A2 showed a significant success rate: 86%. This rate remained however low for strategy C2 (genotyping of founders animals without testing of culled animals: 38%). Diagnostic-oriented strategies always failed.

### Duration of the qualification process

**Figure 1:**
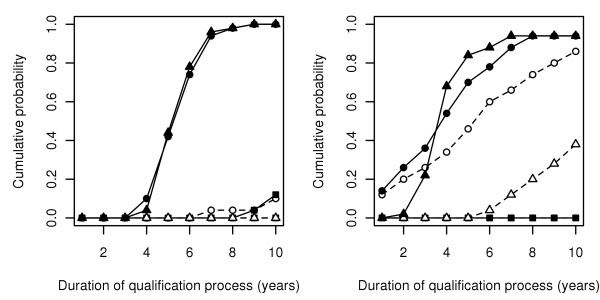
**Duration of the qualification process for substantiating scrapie freedom**. Results obtained in simulated flocks from a susceptible (left: 30% susceptibility alleles and 10% resistance alleles, 50 flocks) and from a resistant population (right: 10% susceptibility alleles and 30% resistance alleles, 50 flocks) according to the qualification strategy: (i) full genotyping (circles) with (A1: solid line, filled symbol) or without (A2: dashed line, hollow symbol) negative test results for culled animals, (ii) genotyping and negative test result for each culled animal (squares) with (B1: solid line, filled symbol) or without (B2: dashed line, hollow symbol) known flock pedigree, and (iii) genotyping of founders animals with known flock pedigree (triangles) with (C1: solid line, filled symbol) or without (C2: dashed line, hollow symbol) negative test result for culled animals). The design prevalence is 1%.

In a susceptible population, strategies A1 and C1 were the only strategies for which high success rates were achieved. Qualification successes were obtained between the 4^th ^and the 9^th ^year (Figure 1, left), with an mean value of 6 years (Table 1). Distributions of the qualification process duration were very close (Figure 1, left), and in 62% of the 50 simulated flocks, both durations were identical. Absolute difference was ≤1 year in 96% of simulated flocks.

In a resistant population, mean duration of the qualification process was 4 years with strategies A1 and C1, *i.e*. 2 years shorter than in a susceptible population. Distributions of the qualification process duration (Figure 1, right) showed that, if average durations are identical, the earliest successes were obtained with strategy A1: qualification succeeded at the 1^st ^simulated year in approximately 15% of simulated flocks, duration being ≤ 2 years in about one quarter of the simulated flocks. Conversely, with strategy C1, no qualification success was obtained at the 1^st ^year, and very few at the the 2^nd ^year. For both strategies, the longest observed durations were 7 years. Finally, with strategy A2, average qualification was 1 year longer than with strategies A1 and C1 (Table 1). Distribution of qualification durations (Figure 1, right) appeared comparable to the distribution obtained for A1, the final proportion of qualification successes being lower.

### Costs of qualification process

**Figure 2:**
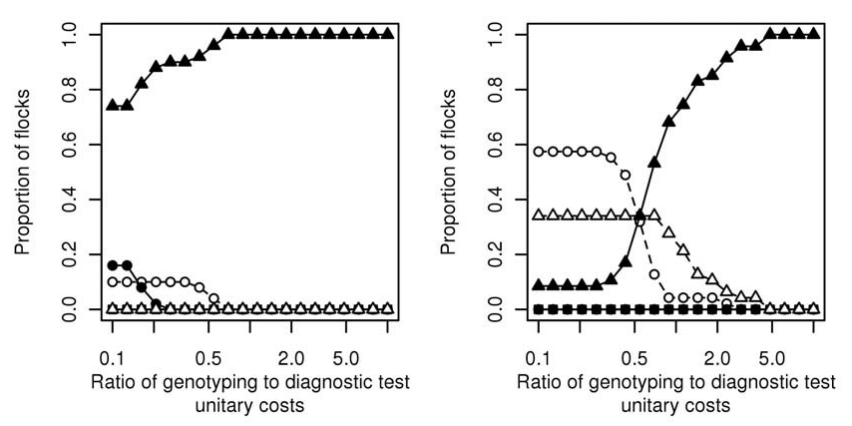
**Qualification strategy with the lowest associated cost according to the genotyping/diagnostic test unitary costs ratio**. Results obtained in simulated flocks from a susceptible (left: 30% susceptibility alleles and 10% resistance alleles, 50 flocks) and from a resistant population (right: 10% susceptibility alleles and 30% resistance alleles, 50 flocks) according to the qualification strategy: (i) full genotyping (circles) with (A1: solid line, filled symbol) or without (A2: dashed line, hollow symbol) negative test results for culled animals, (ii) genotyping and negative test result for each culled animal (squares) with (B1: solid line, filled symbol) or without (B2: dashed line, hollow symbol) known flock pedigree, and (iii) genotyping of founders animals with known flock pedigree (triangles) with (C1: solid line, filled symbol) or without (C2: dashed line, hollow symbol) negative test result for culled animals). The design prevalence is 1%.

In a susceptible population, with strategies A1 and C1, approximately 200 diagnostic tests were performed, on average, before qualification succeeded. With strategy C1, the number of genotyping exams was however much lower than with strategy A1 (Table 1). The strategy with the lowest cost was thus strategy C1, whatever the genotyping/diagnostic test unitary costs ratio (Figure 2, left). In particular, even if the cost of a genotyping exam is very low (ratio of 0.1), strategy C1 remained the strategy with the lowest cost in approximately 3/4 of simulated flocks.

In a resistant population similar results were obtained when comparing average costs with strategies A1 and C1 (Table 1): average number of diagnostic tests were very close (140 tests) but the average number of genotyping exams was 3 times higher with strategy A1. Strategy A2 (full genotyping without testing of culled animals) represented however a possible alternative for which the number of genotyping exams was close to that performed with strategy A1, but for which no diagnostic test was performed. The identity of the strategy with the lowest associated cost was thus more variable when the genotyping/diagnostic test unitary costs ratio changed (Figure 2, right). If the cost of a genotyping exam was less than half that of a diagnostic test, the strategy with the lowest associated cost was A2. In particular, if the unitary cost of genotyping exams was very low (ratio of 0.1), strategy A2 was the less costly strategy for 60% of simulated flocks. Conversely, if the cost of a genotyping exam was more than half that of a diagnostic test, the strategy with the lowest associated cost was C1. Strategy C2 had an intermediate position between A2 and C1, but its success rate was low.

## Discussion

Most of the methods for demonstrating the absence of a transmissible disease have been developed at the zone or the country level, but they can be applied at the flock level. These methods are entirely or partly based upon random surveys in the studied population, designed to detect the disease if it is present at a given level (the design prevalence). If the survey fails to detect any infected animal, one can state with a certain probability that the disease level is below the design prevalence, and, in practice, the population is considered disease-free. Several studies have been published for computing sample size considering either a diagnostic test of perfect sensitivity and specificity [17], or an imperfect diagnostic test [18,19].

A common assumption of the preceding approaches is that, when the disease is present, all the tested animals have the same probability to be found infected. This assumption is not always the case. Several tests with different sensitivities may be used on different animals [20]. Individual variations of test sensitivity may also exist. For BSE, as infection evolves slowly in the infected animals, the probability for an infected animal to be test-positive increases with age [21,22]. These variations may be taken into account by weighting each negative diagnostic result by the corresponding sensitivity, considering both individual sensitivity variations and tests sensitivity variations. For example, BSE negative test results obtained from old animals (for which sensitivity is high) bring more evidence of disease freedom than negative test results obtained from young animals (for which sensitivity is low). This approach may be adapted to scrapie, considering genetic variations of susceptibility as individual variations of the probability to detect the disease when a flock is infected. Negative test results obtained in animals of susceptible genotypes should then bring more evidence of disease freedom than negative test results obtained in animals of resistant genotypes. However, this leads to a paradoxical situation in which, for substantiating disease freedom, a greater amount of testing is needed in resistant flocks than in susceptible flocks, despite the fact that the probability of disease freedom is *a priori *higher in resistant flocks than in susceptible flocks.

To solve this paradox, a possible approach would be to combine random survey results with another source of data aiming at evaluating overall flock susceptibility to infection (*i.e*. the probability that, if introduced in the flock, disease agent would circulate). A flock would then be considered scrapie-free either on the basis of a very low flock susceptibility level (most or all the animals being of resistant genotypes), or on the basis of the negative results of a random survey, or on the basis of a combination of the two preceding data sources using methods such as stochastic scenario trees [23]. However, instead of considering two separate sources of evidence for scrapie freedom, we chose to adapt the probabilistic framework in order to solve the preceding paradox. The proposed approach may be used for finite populations, when individual susceptibility to infection can be considered as being binary, and when individual characteristics allow to compute, for each animal of the flock, the probability to be susceptible to infection. The survey aiming at detecting disease is then modelled as an hypergeometric process that allows taking into account both the flock susceptibility (the number of susceptible animals) and the amount of testing done. In particular, the proposed approach accounts for the fact that if the flock susceptibility level is low (most of the animals belonging to resistant genotypes), the probability that the number of susceptible animals is below the design prevalence may be high enough for substantiating scrapie freedom without performing any diagnostic test.

Several qualification strategies are proposed for demonstrating a flock is free from scrapie (design prevalence: 1%): genotyping-oriented strategies, diagnostic-oriented strategies and pedigree-oriented strategies. Results show that genetic data play a central role for substantiating freedom from scrapie, as the diagnostic-oriented strategies fail in most cases. Thus performing diagnostic tests and genotyping exams on all culled animals does not allow demonstrating scrapie absence within a reasonable time horizon (10 years). Oppositely, two strategies show close and satisfactory performances: a genotyping-oriented strategy in which all the flock animals are genotyped and a pedigree-oriented strategy in which only founders animals are genotyped, the flock pedigree being known. In both cases, diagnostic tests are performed on culled animals. These two strategies are successful in the vast majority of cases. Four years on average are required to demonstrate disease freedom in a resistant population (30% of resistance alleles, 10% susceptibility alleles), and six years in a susceptible population (10% of resistance alleles, 30% susceptibility alleles). The pedigree-oriented strategy is however the less costly and, more generally, results show that knowing the flock pedigree always brings a significant added-value, as it allows reducing the amount of genotyping exams. If not routinely recorded, documenting the flock pedigree should thus be encouraged. In the proposed approach, knowledge of flock pedigree is assumed perfect; the model can however be easily modified to take into account incomplete or imprecise knowledge of flock pedigree.

Global cost for substantiating freedom from scrapie depends upon the unitary costs of genotyping exams and of diagnostic tests. Considering a reasonable domain for the ratio between these two unitary costs (from 1/10 to 10) allows to show that, in a susceptible population, a dominant strategy exists: the pedigree-oriented strategy described above is the less costly in the vast majority of cases. The situation is less clear in a resistant population. A genotyping-oriented strategy, solely based upon the realization of genotyping exams on all the flock animals, becomes a valuable alternative to the preceding pedigree-oriented strategy. In particular, when the cost of a genotyping exam is less than half the cost of a diagnostic test (which corresponds to the present situation in France), this genotyping-oriented strategy becomes the lowest-cost strategy.

## Conclusion

While performed for a single flock type and two genetic contexts, our study shows that it is possible to demonstrate that scrapie is absent from a flock. However, there is no general strategy that would always minimize the costs, and in practice, the choice of a qualification strategy should be adapted to genetic conditions (e.g. average resistance level in the breed), as well as to the respective costs of diagnostic tests and genotyping exams. The duration before scrapie freedom is demonstrated remains long (except for flocks with a high genetic resistance level) and lasts several years.

Nevertheless, qualification schemes could be useful tools for scrapie control programmes. In the case of voluntary control programmes, the practical interest of such schemes remains to be investigated, by comparing potential benefits brought by a qualification (added value of the produced animals) with costs induced by qualification procedure. However, if a circulation of BSE agent in sheep flock were demonstrated, scrapie control programmes would probably quickly become mandatory (or strongly encouraged through trade restrictions). As for other infectious diseases, qualification schemes could then be used to classify flocks according to their status, a classification upon which control measures could be based.

## Methods

### Substantiating freedom of a genetically controlled infectious disease

Posit a flock composed of *N *animals numbered 1-*N*, of which a subset *J *has been tested negative for infection detection. Test sensitivity and specificity are assumed perfect. We consider a binary model for individual susceptibility: either an animal is fully susceptible to infection, or it is refractory and can not be infected (or would then clear quickly the infectious agent). Let *s*_*i *_be the susceptibility status of the *i*th animal: *s*_*i *_= 0 if animal *i *is refractory and *s*_*i *_= 1 otherwise.

We define the posterior distribution of within-herd prevalence, denoted *d *(the number of infected animals), conditional to the the fact that all the test results obtained in the animals of *J *were negative:

(1)

where:

- ⟨0,1⟩^*N *^represents the set of all the possible *N*-length sequences composed of zeros and ones and *X*_*i *_is the *i*th element of sequence *X*,

- *P*(*d *= *D*|*M*, *m*) is the posterior probability of prevalence, given the number *M *of fully susceptible animals in the flock and the number *m *of fully susceptible animals among those tested, all of which were found uninfected.

If the tested animals are randomly chosen, the number of infected animals in the sample *J *results from an hypergeometric process: *m *animals are randomly picked without replacement from a group of *M *animals of which *D *are infected, and each of these *m *animals turns out to be uninfected. We suppose that no information is available about infection prevalence within the flock. An uniform prior is thus used and *P*(*d *= *D*|*M*, *m*) is [24]:

(2)

Now we assume that the control of individual susceptibility to infection is genetic. The probability *P*(*s*_*i *_= 1) thus depends on the genotype of animal *i*, denoted *g*_*i*_. Posit a set of *n *possible genotypes ranked by increasing susceptibility: *G*_1 _corresponds to the most resistant genotype, while *G*_*n *_corresponds to the most susceptible one. Genotype-specific susceptibility is modelled by the probability, for an animal of a given genotype, to be fully susceptible to infection. All the animals of the most susceptible genotype *G*_*n *_are assumed fully susceptible to infection. For the other genotypes, this probability is measured by the relative risk of infection *RR*_*x*_, for animals of genotype *G*_*x *_taking as a reference the animals of the most susceptible genotype *G*_*n*_:

(3)

The overall probability for an animal to be fully susceptible to infection is then the mean of the genotype-specific relative risks, weighted by the probability, for the animal, to harbour the specified genotype:

(4)

Similarly, the overall probability for an animal to be refractory to infection is:

(5)

Equations (4) and (5) can be combined:

(6)

Substituting the preceding expression in equation (1) finally gives:

(7)

Exact computation of equation (7) has a complexity proportional to *N*^*n*^, which is not computationally tractable except for very small values of *N *and *n*. Therefore a Monte-Carlo procedure is used to estimate the posterior probability distribution for the number of infected animals in the flock. The susceptibility status of each animal is randomly set under the probability (8), and equation (2) is then used to generate a posterior probability distribution. Both operations are repeated 10^3 ^times. The estimate of the posterior probability distribution of within-flock prevalence is the average of these 10^3 ^distributions.

(8)

The posterior distribution of prevalence is finally used to compute the probability that the negative test results observed in sample *J *would be achieved if the disease were present at a specified level: the design prevalence [20], denoted *π*:

(9)

### Combining data sources for substantiating disease freedom

The above approach for estimating the posterior probability distribution of prevalence is based upon two flock-specific data sources: the set *J *of tested animals, and the genotype-specific probabilities *P*(*g*_*i *_= *G*_*x*_), for each individual *i*, to harbour genotype *x*.

The set of tested animals is an input data directly defined by some testing scheme. Note that the above approach allows deriving a posterior distribution of *d*, even if no animal is tested for infection detection (*J *is empty). In this case, the result is the distribution of the number of fully susceptible animals in the flock. If, according to this distribution, the proportion of fully susceptible animals is lower than the design prevalence (at a predefined confidence level), the flock may be considered scrapie free without performing any test.

Two kinds of data may be used to set the genotype-specific probabilities: results of genotyping exams, and pedigree data that correspond to the parent-child relationships between the flock animals. The three following rules are used to set *P*(*g*_*i *_= *G*_*x*_) for each individual:

(1) If animal *i *has been genotyped, we set *P*(*g*_*i *_= *γ*) = 1 and 0 for the genotypes other than *γ*, where *γ *is the actual genotype of *i *(genotyping errors are neglected).

(2) If animal *i *has not been genotyped and if the flock pedigree is unknown, some default genotype-specific probabilities must be used. A first approach would be to use the distribution of genotypes in the population to which the flock belongs (e.g. the sheep breed population). However this is not consistent with a context of qualification where the goal is to bring convincing elements showing that the infection is not present in a specific flock (not in an average flock). Default genotype-associated probabilities are thus set assuming the worst case scenario. If animal *i *has been tested for infection detection (*i *∈ *J*), the default value is the most resistant genotype: *P*(*g*_*i *_= *G*_1_) = 1 and 0 for the genotypes other than *G*_1_. Conversely, if animal *i *has not been tested, default value is the most susceptible genotype: *P*(*g*_*i *_= *G*_*n*_) = 1 and 0 for the genotypes other than *G*_*n*_.

(3) If animal *i *has not been genotyped and if the flock pedigree is known, the latter is used to compute the genotype-associated probabilities. Pedigree analysis methods allow to derive, from partial genotyping data, genotype-associated probabilities for each of the individuals of a pedigree. Exact probabilities may be computed for simple pedigrees using algorithms known as peeling algorithms [25]. When the pedigree contains loops (e.g. when a ram is crossed with one of its daughters, which is common), only estimates can be obtained using iterative algorithms [26,27]. We use the iterative algorithm proposed by Thallman et al. for large pedigrees [28,29]. This implies to set the genotype-associated probabilities for founders animals. For non-genotyped founders animals, the rule (2) is applied, whereas for the genotyped animals rule (1) is applied.

### Comparing qualification strategies in simulated flocks

#### Random generation of flock genetic trajectories

Qualification strategies were compared in simulated flocks, the genetic trajectories of which were randomly generated. We used a simple individual-centered simulation program in which each animal was represented by a unique id, its genotype, sex, year of birth, and the couple of ids of its parents. Time was discrete with a yearly time step. Each simulated year, the program simulated the culling of ewes and rams followed by the purchase of renewal rams and the birth of lambs of which some were kept as renewal animals.

Yearly ewes culling risk was supposed to increase linearly with age, and age at cull thus followed a Weibull distribution with a shape parameter of 2 and a scale parameter of 4 *E*_*e*_/*Γ*(3/2) (where *E*_*e *_is ewes life expectancy and *Γ *is the Gamma function) [30]. This distribution was used to choose randomly, at each time step, the list of the culled ewes. Culled rams were randomly chosen under a probability of 1/*E*_*r*_, where *E*_*r *_is the average residence time of a ram in a given flock.

Flock renewal aimed at maintaining flock size at a constant level. We assumed that renewal rams were always purchased. Their genotype was randomly chosen from the distribution of genotypes in the population to which the flock belongs denoted  (excluding the most susceptible *G*_*n *_rams when the flock was involved in a qualification process). We assumed the flock closed for ewes renewal. For each renewal female lamb, the parents ids were randomly chosen among ewes and rams. The genotypes of both parents then allowed choosing randomly the genotype of the product according to Mendel's laws.

#### Parameterization and exploitation

Simulated flocks had a medium size, with 150 ewes and 4 rams. For ewes, we assumed a life expectancy *E*_*e *_= 4 years and a maximal age of 10 years. Rams were kept, on average, *E*_*r *_= 2 years. Three allelic classes were considered (susceptibility alleles, intermediate alleles and resistance alleles) [31]. They defined *n *= 6 genotypes, from *G*_1 _(the most resistant genotype) to *G*_6 _(the most susceptible genotype). Genotype-specific relative risks of infection decreased linearly on a log_10 _scale, from *RR*_6 _= 1 to *RR*_1 _= 10^-5^.

Two distinct contexts were considered for the genotypes distribution in the population :

- a susceptible population, with 30% of susceptibility alleles and 10% of resistance alleles,

- a resistant population, with 10% of susceptibility alleles, and 30% of resistance alleles.

Qualification strategies were compared in 50 randomly generated flocks for each of the two preceding populations. Initial flock composition was randomly generated from . At each simulated year, the six qualification strategies were successively simulated to compute the six corresponding posterior prevalence distributions. Time horizon was 10 years. Prevalence distributions were then used to calculate for each simulated year and qualification strategy, the probability that infection prevalence was below the design prevalence (Eq. 9).

Sensitivity and specificity of scrapie diagnostic tests were assumed perfect. This assumption allowed to simplify the probabilistic model and its parameterization. However, it is known that the tests routinely used for scrapie diagnostic have a limited sensitivity, due to the nature of the biological sample (obex). A recent study [32] showed that, in an affected flock, 40 animals were obex-positive while 13 were obex-negative but positive for samples from lymphoreticular system. This corresponds to a relative sensitivity of 75% for rapid tests on obex, taking as a reference the joint results of rapid tests on obex and on samples from lymphoreticular system. To compensate for this non-perfect sensitivity, a low value of 1% was chosen for the design prevalence, below the range of 5–30% reported in published studies [32].

Strategies were first compared in terms of success/failure. Qualification process failed if, after 10 simulated years, the probability that infection prevalence is below the design prevalence remained <95%. Conversely, if this probability was ≥95% at the 10^th ^year or before, qualification process was considered to be successful, and ended. For each population, proportions of successes associated to each strategy were computed and compared. Qualification successes may be obtained more or less early before the 10^th ^year. When qualification process succeeded, its duration (number of years) was calculated. For each population, strategy-specific distributions of this duration were computed and compared.

Finally, besides the duration of the qualification process, the associated costs may be computed in terms of numbers of diagnostic tests and genotyping exams that have been performed. Strategies defined above use preferentially either diagnostic tests, or genotyping exams, or both. Actual costs will depend on the corresponding unitary costs. Instead of fixing these arbitrarily, we considered their ratio that allowed us to determine, for a specific flock, the strategy with the lowest associated cost. Fixing the value of the genotyping/diagnostic test unitary cost ratio, it was possible to compute, for each population, the probability that a given strategy was the lowest cost strategy (*i.e*. the proportion of the simulated flocks for which this strategy had the lowest associated cost). These strategy-associated probabilities were computed for varying unitary costs ratios (between 0.1 and 10).

## Authors' contributions

BD participated in the design of the project, constructed the model, conducted the simulations, performed the statistical analysis and drafted the manuscript. MJM, CD and DC participated in the design of the project, in the parameterization of the model, in the analysis of the simulations results, and in the redaction of the manuscript. All the authors read, edited and approved the final version of the manuscript.
